# International financial institutions and human rights: implications for public health

**DOI:** 10.1186/s40985-017-0074-3

**Published:** 2017-11-30

**Authors:** Thomas Stubbs, Alexander Kentikelenis

**Affiliations:** 1 Centre for Business Research, University of Cambridge, Cambridge, UK; 2 Department of Politics & International Relations, Royal Holloway, University of London, Egham, UK; 3 Trinity College, University of Oxford, Oxford, UK; 4 Department of Sociology, University of Amsterdam, Amsterdam, The Netherlands

**Keywords:** Structural adjustment, Human rights, International financial institutions, International Monetary Fund, World Bank

## Abstract

Serving as lender of last resort to countries experiencing unsustainable levels of public debt, international financial institutions have attracted intense controversy over the past decades, exemplified most recently by the popular discontent expressed in Eurozone countries following several rounds of austerity measures. In exchange for access to financial assistance, borrowing countries must settle on a list of often painful policy reforms that are aimed at balancing the budget. This practice has afforded international financial institutions substantial policy influence on governments throughout the world and in a wide array of policy areas of direct bearing on human rights. This article reviews the consequences of policy reforms mandated by international financial institutions on the enjoyment of human rights, focusing on the International Monetary Fund and World Bank. It finds that these reforms undermine the enjoyment of health rights, labour rights, and civil and political rights, all of which have deleterious implications for public health. The evidence suggests that for human rights commitments to be met, a fundamental reorientation of international financial institutions’ activities will be necessary.

## Background

After the 2007 global financial crisis, several European countries experienced growing public debts and shrinking economic output. Greece was one of several countries that, in 2010, sought the assistance of international financial institutions to finance upcoming debt repayments. The country initially borrowed €110 billion from the International Monetary Fund (IMF) and Eurozone partners under strict condition that there would be drastic curtailing of government spending. This had deleterious and potentially long-lasting public health implications. Cuts to municipal budgets led to scaling back of mosquito-spraying programmes, resulting in the re-emergence of locally transmitted malaria for the first time in 40 years; public hospital budgets were reduced by 26%, leading to staff overwork, increasing waiting lists, and shortages of medicines and medical equipment; and prevention and treatment programmes for illicit drug use also faced cuts, leading to increases in HIV infections from intravenous drug users [1–3]. In the throes of unprecedented levels of unemployment, welfare services were also cut in Greece, leaving vulnerable groups without access to income and confronted with an overburdened and increasingly pricey health system [4]. In a scathing assessment of the policy reforms adopted at the behest of the IMF and Eurozone partners, the United Nations Human Rights Committee concluded that they were in violation of several human rights obligations, including the right to work, health, and social security [5, 6].

The experience of Greece is far from unique. International financial institutions (IFIs)—a term collectively referring to the IMF, the World Bank, and regional development banks—condition the provision of loans, grants, and debt relief on the implementation, by the recipient country, of a series of policy reforms. These are typically aimed at making the fiscal and debt situation sustainable, improving competitiveness, and boosting economic growth. Common measures that governments are required to implement include privatisation of public assets, public spending cuts (or ‘austerity’), and structural reforms (such as changes to labour market reforms, trade liberalisation, and legal reform). This practice of ‘conditionality’ affords IFIs substantial policy influence on governments throughout the world, thereby reducing national policy space and undermining national development agendas [7]. The measures also have implications for the enjoyment of human rights, including the right to the highest attainable standard of health.

This article provides an overview of these policies and their impact on the ability of implementing governments to provide basic public services necessary for the realisation of human rights. First, we discuss the mandates, priorities, and lending practices of the two most influential and well-resourced IFIs, the IMF and the World Bank. Subsequently, we review existing debates on the effects of conditionality for the enjoyment of human rights, organised into three thematic dimensions: health, labour, and civil and political rights. We conclude by discussing the broader implications for public health and offer suggestions for revamping IFI lending practices in ways that ensure respect for human rights.

### International financial institutions in historical context

IFIs, such as the IMF, World Bank, and regional development banks, are considered the world’s most powerful agents of economic reform [8, 9]. In the past four decades, they have set the fiscal parameters within which public policies—including health—operate in developing countries via their so-called ‘structural adjustment’ programmes [10]. Among public health advocates, the role of IFIs in affecting population health gained notoriety after the publication of a critical UNICEF report ‘Adjustment with a Human Face’ [11]. This report found adverse child and maternal health outcomes attributable to the policy reforms promoted by these organisations. Scrutiny surrounding the deleterious effects of IFI activities on the enjoyment of health rights have more recently stepped up following socially regressive programmes in advanced European economies, as the Greek tragedy alluded to in our introduction illustrates.

Serving as lenders of last resort to countries experiencing unsustainable levels of sovereign debt, the IMF and World Bank are among the most influential IFIs [12]. The two organisations possess a similar array of tools for persuading governments to adopt reforms, the best known of which is conditionality: the practice of requiring policy reforms in exchange for access to resources. In conditional lending arrangements with IFIs, policy reforms are outlined in documents specifying timetables for their introduction and are assessed on a regular basis. Non-implementation can result in delays in loan disbursements and—ultimately—the suspension of lending altogether. While this process is typically coercive, there are instances where borrowing governments seek out particular policy reforms in order to overcome domestic political opposition or to use IFIs as political scapegoats when establishing unpopular policies [13].

The origins of these organisations can be traced to the Bretton Woods conference in July 1944, which laid the foundations of the post-war economic order [8]. In response to appeals for a system of global financial governance and greater international economic cooperation, the IMF and the International Bank for Reconstruction and Development (later known simply as the World Bank) were established soon after in 1945.

Both organisations have diverged from their original mandate set out at Bretton Woods. The role of the IMF was initially to oversee the exchange rates of member governments and to make financial resources available to member governments facing balance of payment problems [14]. Following the shift to floating exchange rates in 1973, only the second—and most controversial—aspect of the IMF’s mandate survived. For its part, the World Bank was established to provide investment capital for post-war reconstruction and economic development. It initially specialised in lending to advanced nations for infrastructural projects, such as ports, railroads, and hydroelectric dams. In response to demands of developing countries for greater financing, however, world leaders set up an additional organisation within the World Bank in 1960, the International Development Association (IDA). This shaped the Bank into a more development-focused organisation, and its mandate expanded to encompass the eradication of global poverty [15].

In meeting these—revised—functions, the two organisations became involved in promoting market-liberalising reforms as part of their lending operations [16]. Through the 1970s and 1980s, the IMF introduced a series of lending programmes targeting structural change, such as the Extended Fund Facility and Structural Adjustment Facility [17, 18]. Prior to this, reforms attached to IMF loans were largely confined to a series of standard quantifiable targets, such as ceilings to government expenditure and net domestic assets. The new facilities incorporated detailed micro-economic reforms spanning a wide array of policy areas. For its part, the World Bank continued the tradition of providing ‘project’ lending—for instance, loans to build roads or schools—but, in 1980, also introduced a ‘programme’ lending facility of its own, the Structural Adjustment Loan [8]. As the 1980s unfolded, the World Bank steadily devoted a larger proportion of its resources to programme lending than to development projects, while the IMF became—for all intents and purposes—a development institute that collaborated with the World Bank [16, 17].

Against a background of debt crises, structural adjustment programmes became ubiquitous in the 1980s, achieving notoriety for requiring low- and middle-income countries to implement free-market policies [8]. Indeed, the term ‘structural adjustment’ became shorthand for an extensive range of reforms designed to promote fundamental, comprehensive, and enduring overhaul of a borrowing country’s policy arrangements [18]. These reforms entailed four key policy pillars: stabilisation, liberalisation, deregulation, and privatisation [19]. Stabilisation refers to fiscal deficit reduction measures—via revenue generation and government expenditure cuts (‘austerity’)—and to money supply reduction measures, the intent of which is to control inflation, stabilise currencies, and free resources to repay external debt. Liberalisation entails the elimination of barriers to trade and the movement of capital in order to facilitate access to international markets and promote foreign direct investment. Deregulation involves the repeal of government rules, regulations, checks, and balances surrounding economic activity—such as industry entry criteria—geared towards abolishing perceived inefficiencies to the functioning of the private sector. Finally, privatisation occasions the selling of state-owned enterprises and natural resources to the private sector, with the aim of improving the economic management of these industries.

For both organisations, the practice of conditionality became a prominent feature of their *modus operandi* [20], and—by the 1990s—was a staple vehicle for implementing the transition to capitalism in post-communist countries. Securing compliance with these prescriptions was achieved not only through the threat of suspending the loan from borrowing governments but was also encouraged through closer relationships between the IMF, World Bank, various regional development banks, and private creditors [16]. In particular, the various multilateral agencies harmonised and upheld one another’s reforms, while the presence of structural adjustment programmes also served as a ‘stamp of approval’ that could catalyse additional bilateral aid from donor governments—including debt relief—and mobilise financial flows from private international capital [21, 22]. Among the World Bank and regional development banks, policy leverage was also consolidated through greater selectivity in awarding project loans to countries that were compliant with these policies [23].

The IMF and World Bank were widely criticized in subsequent years, especially following their mishandling of the financial crises in Mexico, East Asia, Russia, and Argentina [24, 25]. The organisations promised that painful austerity measures would be justified in the long run by sustained economic growth. Yet, economic growth failed to materialise [26], while the negative consequences for the enjoyment of human rights mounted (discussed below). Faced with disconfirming evidence, the view of the organisations was that structural adjustment programmes had paid insufficient attention to the institutions that allow markets to function, such as laws and judicial systems, but that the underlying market-liberalising impetus was essentially correct [8].

By the early 2000s, in response to extensive criticisms, the IMF and World Bank pledged to strengthen the pro-poor orientation of their programmes and to allow for flexible policy design [27, 28]. And, in 2009, following the onset of the sovereign debt crises in Europe, structural adjustment programmes of the IMF—in collaboration with European Union institutions—spread to the advanced European economies of Greece, Iceland, Ireland, Portugal, and Cyprus. These programmes were similar to those advocated by the IMF and the World Bank in low- and middle-income countries insofar as they relied on extensive market-liberalising conditionality [29, 30].

### Methodological approach

The main objective of this article is to review the human rights implications of IFI-administered structural adjustment programmes. The review incorporates literature on both developing and advanced nations. We focus on the IMF and the World Bank because they are widely considered the most influential of IFIs [12]. While regional development banks also administer structural adjustment, these bear little point of distinction from those of the IMF and World Bank, who often co-finance their loans and share expertise [10].

We conducted a comprehensive search of the literature covering structural adjustment and human rights. First, we searched the websites of the two organisations under review, as well as secondary literature, for information on their human rights stance, related activities in the field, and internal research. Second, we searched two electronic databases (PubMed/MEDLINE and Web of Science) for academic literature. The database search was conducted on February 2017. The search terms included the name of each organisation reviewed or related terminology (‘World Bank’, ‘Fund’, ‘IMF’, ‘structural adjustment’, ‘polic*’, ‘reform*’, ‘condition*’), as well as human rights-related terms (‘right*’, ‘health’, ‘labour’, ‘labor’, ‘democ*’, ‘social’). Third, we searched for grey literature on the topic using Google and Google Scholar, using an equivalent methodology to the database search outlined above. Fourth, we scanned reference lists of the materials obtained.

The method used to examine the evidence obtained was a narrative synthesis, in order to map proximal and intermediate pathways and identify hypotheses. This approach was appropriate given that data are limited and often haphazardly situated in a large corpus of literature. For example, some studies link the incidence of structural adjustment with the infringement of health and labour rights, whereas other studies document how the latter results in a rise of social protect and instability, leading to violent bouts of repression by the ruling government that amounts to breaches of civil and political rights. We synthesise such findings in order to yield nuanced understandings of the relationships of interest, organised into three human rights thematics: health rights, labour rights, and civil and political rights. These all have broader implications for public health, an issue we return to in the concluding section.

## Results

### Health rights

The realisation of the right to health is codified in the 1966 International Covenant on Economic, Social, and Cultural Rights (ICESCR) and expanded upon in General Comment 14 issued by the UN Committee on Economic, Social and Cultural Rights in 2000 [31]. Rather than a minimalist agenda encompassing health interventions for the most vulnerable, the right to healthcare in ICESCR forms a comprehensive framework of minimal standards and progressive realisation towards the highest attainable standard of health. It offers a legitimate claim to freedoms and entitlements directly related to health, including access to quality and affordable healthcare, as well as indirectly related to health, such as access to food, water, employment, and education [32]. The ICESCR and the General Comment also enshrines these rights as national and international obligations, with the latter related to development cooperation for health [32]. Although the IMF and World Bank have long claimed that they are concerned about the health consequences of their policy advice [7, 33], the available evidence suggests that many loan reforms breach these obligations and are detrimental to health outcomes.

IMF and World Bank conditionality *directly* impacts upon the fulfilment of the right to health by prompting changes to the volume and quality of services provided (like health facilities and medical supplies) [30]. On the one hand, to shelter sensitive expenditure from austerity measures, structural adjustment programmes incorporate poverty reduction reforms, typically in the form of priority spending floors that stipulate minimum expenditures on health and education [34–37]. Indeed, the past decade has seen a rapid rise in the inclusion of poverty-reduction reforms [7]. These may in part explain findings from cross-national studies showing that structural adjustment programmes are associated with increases in social spending in sub-Saharan African low-income countries, autocratic countries, and Latin American countries [38–40]. Archival evidence on IMF programmes in West African nations also demonstrates that, in select instances, priority spending floors contributed to increases in budgetary allocations for health [41, 42].

On the other hand, there is compelling evidence to suggest that poverty reduction is accorded subsidiary importance to fiscal reforms. Priority spending targets are most often non-binding reforms that serve as markers for broader progress assessment, but do not automatically suspend a programme if unmet; whereas fiscal deficit targets are binding and automatically suspend the loan [43]. Furthermore, research shows that priority spending targets were observed only about half of the time, even though fiscal deficit reforms were almost always met [7, 41, 44]. As part of an austerity drive, governments may be under pressure to cut social spending in order to meet fiscal deficit targets, which can—in turn—limit the accessibility and affordability of healthcare [7, 10, 18, 41, 45–47].

Several cross-national studies on the effects of structural adjustment on social expenditure suggest that it is associated with decreases in spending in low-income countries outside sub-Saharan Africa and democratic states [38, 39]. A recent study focusing on West African countries also found that each additional IMF condition reduces government health expenditure per capita by 0.25% [41, 42]. Consequently, countries experience medical supply shortages and replacement of defunded health services with ineffective traditional programmes [10, 18]. Empirical studies assessing the effect of health expenditures or government spending more broadly find a significant and detrimental relationship with infant mortality, under-five mortality and several other health outcomes [10, 48, 49]. The UN Human Rights Council also reports that declines in government spending promoted by IMF programmes in Cyprus, Greece, and Ireland translated into decreases in healthcare staff, reductions in the number of hospital beds, and increases in waiting times for medical procedures [6].

Aside from fiscal reforms, institutional reforms included in structural adjustment programmes can also limit the accessibility and affordability of healthcare. One common measure has been the introduction of user fees for access to healthcare and co-payments for medicines or services [50, 51]. The rationale for applying such fees was generating additional resources, improving efficiency, and increasing access [52]. In practice, they have undermined the right to health for poor and vulnerable people, both in terms of reducing their use of such services due to prohibitive costs and through impoverishment by the effects of unavoidable health expenses [53–55]. A design simulation model of 20 African countries employing user fees for health concluded that abolition of fees could prevent an estimated 233,000 under-five deaths annually, or 6.3% of such deaths in these settings [54]. In recent years, the IMF and World Bank policy consensus vis-à-vis low-income countries has moved away from reliance on user fees. Yet, recent structural adjustment programmes in Europe advocated the introduction or increase of user fees or co-payments and made eligibility criteria for subsidised health services more stringent [1, 2, 4, 6, 10, 56, 57].

Institutional reforms calling for deregulation of the health sector have also enhanced the private sector’s role in healthcare provision [58]. While those able to afford it can obtain access to a broader bundle of services, this can be coupled with austerity-driven roll-backs of government health provision to a more limited array of services, or outsourcing to non-governmental organisations who are often less-equipped to provide comprehensive and high-quality health services [18, 46, 59–62]. Furthermore, structural adjustment programmes are linked to health system decentralisation; that is, transferring fiscal and operational responsibilities to the subnational level [41, 44, 58]. In principle, decentralisation can make health systems more responsive to local needs; but—in practice—they often create governance problems and exacerbate local institutional weaknesses that undermine the right to health, especially when managing nationwide disease outbreaks [41, 44, 63].

Labour-related reforms can also impact upon the right to health in terms of the quantity and quality of the public sector health workforce available, via redundancies, hiring freezes, or wage cuts [41, 44, 64–66]. In West Africa alone, almost half of all years with IMF programmes included reforms stipulating layoffs or caps on public-sector recruitment and limits to the wage bill between 1995 and 2014 [41]. These targets can impede a country’s ability to hire, adequately remunerate, or retain health-care professionals, and are linked to medical ‘brain-drain’ as healthcare workers migrate in search of better employment opportunities [67, 68]. The IMF reports that such wage caps have been discontinued in their programmes [69], but recent evidence shows they are still incorporated into some [7].

Notwithstanding these direct effects of conditionality, the right to health can also be *indirectly* affected. First, currency devaluations—advocated by the IMF and World Bank to improve the external competitiveness of countries—impede access to imported medicines and medical equipment by raising the price of imports [70]. Second, removal of customs duties reduces trade tax revenues in the short-run, which—unless replaced by alternative sources of revenue—can undermine the fiscal basis of health policy; however, if the economic benefits of openness stimulate economic growth in the medium-term or if tax revenues are raised elsewhere (e.g. via new consumption taxes or improved tax collection), then greater public revenues could be invested in health [71, 72]. Third, privatisation of state-owned enterprises—designed to raise funds for cash-strapped governments—can result in the medium and long-run to losses in reliable public revenue sources for the state to fund health; where state-owned enterprises provide health coverage to employees, these benefits may be withdrawn post-privatisation, resulting in loss of access to healthcare [73–76]. Fourth, conditionality may be interpreted by donors as firm commitment to reform, rewarded with increases in aid that offset lost revenues for health elsewhere; even so, evidence suggests that aid substitutes—rather than complements—government spending on health, and that aid flows increase for general budget support and debt relief but not for health [22, 77, 78].

Shifting further down the causal chain to the ‘social determinants’ of health [79], reforms attached to structural adjustment programmes are—fifth—linked to dwindling incomes and increases in unemployment, poverty, and inequality, which are—in turn—root causes of a cascade of health problems over the life-course [6, 80–82]. For instance, wage caps and privatisations can result in job losses, which are linked to increases in alcoholism and suicide in post-communist European countries; exchange rate liberalisation can increase food prices, which is tied to deteriorations in children’s nutritional status; and the introduction of regressive forms of taxation can reduce poor households’ incomes and thus their ability to afford healthcare or lead healthy lives [76, 83–87]. Sixth, education is another key social determinant of health, as it increases individuals’ knowledge about health and improves social mobility opportunities which—in turn—affect employment and poverty; yet, studies show IMF and World Bank-mandated user fees for primary education impede educational attainment for children, and that structural adjustment also decrease the protective effect of parents’ education on child health [30, 83, 84]. Finally, reforms on environmental policies—typically including deregulation or privatisation of water and sanitation, agriculture, energy, and other natural resources—are linked to environmental degradation, which in-turn affects population health [88–90].

### Labour rights

Several international human rights instruments contain provisions concerning labour rights. The 1966 International Covenant on Civil and Political Rights (ICCPR) articulates the rights to freedom from forced labour and freedom of association, and the ICESCR embodies the right to participate in work and the right to just working conditions. Labour rights are also protected by fundamental conventions of the International Labour Organisation (ILO) and the 1998 ILO Declaration on Fundamental Principles and Rights at Work [91]. In its 2005 General Comment 18 (on the right to work), the UN Committee on Economic, Social, and Cultural Rights understood this as the right of everyone to earn a living by work that is freely chosen, including the right not to be deprived of work unfairly. States are thereby obligated to allocate resources and adopt policies aimed at reducing their unemployment rate [92]. The Committee also stresses that international financial institutions, such as the IMF and World Bank, must pay attention to the protection to the right to work in their lending policies.

The IMF and World Bank have incorporated reforms that *directly* affect the right to work and to just working conditions via the deregulation of labour markets. Their rationale is based on supply-side economics, which posits that firms invest more when labour markets are flexible and when the costs associated with labour protections are low. For example, an IMF staff report on Romania states, ‘[L]abor market rigidities are impediments to a business-friendly environment and Romania stands out compared to other countries, particularly on costs of hiring and firing workers’ [93]. The IMF and World Bank, thus, view labour market flexibility as a key ingredient for enhancing the global competitiveness of goods and services produced in borrowing countries.

However, critics argue that the IMF and World Bank neglect the human rights implications of reducing labour market rigidities [94–97]. In a recent report on structural adjustment programmes, the UN Human Rights Council concludes that such policies have contravened international human rights obligations by eroding labour rights [6]. A study examining 131 countries for the period 1981 to 2003 also found that the more time a country was implementing structural adjustment programmes, the lower the level of protection of labour rights [94]. Another study on 123 countries found a negative association between structural adjustment programmes and collective labour rights, particularly with regard to workers’ freedom of association and the right to collective bargaining both in law and in practice [95].

Labour reforms have been a consistent feature of structural adjustment programmes since the mid-1990s [7]. The majority of these are targets related to wage caps and employment limits which have—in turn—impeded the right to work. Indeed, a study of 110 countries found a negative association between IMF programmes and workers’ wages, as proxied by the labour share of income in the manufacturing sector [82]. A cross-national study examining the effects of IMF-mandated public sector reforms found that governments cut the public sector wage bill only when this is set out as a required condition; but uncovered evidence of backsliding toward higher expenditure on public sector wages once the programme ended [98]. Similarly, the labour share in relation to GDP declined in recent adjustment programmes in Eurozone countries, especially Greece [99].

With regard to employment limits, Moldova’s IMF-designed labour-related reforms included measures to ‘optimize the number of employees in the budgetary sector [… by] eliminat[ing] at least 4,000 positions’ in 2010 [100]. Recently, restrictions on hiring in the public sector were introduced in Cyprus, Greece, Ireland, and Portugal; and Tunisia’s 2014 programme incorporated a salary freeze for civil servants [6]. Studies also link layoffs incorporated in adjustment programmes to declines in unionisation rates (since labour unions are typically more prevalent in the public sector), a weakening of the bargaining power of workers more generally, and an expansion in the informal sector, thereby curtailing the right to decent working conditions [5, 82, 101, 102].

Labour reforms have also featured difficult structural reforms geared towards a fundamental transformation of social security institutions and the deregulation of labour laws. Several studies find that the IMF and World Bank promote labour laws that legalise temporary work contracts, extend probation periods, remove barriers to firing workers, reduce employee entitlements, and dismantle rights to form and join labour unions and to collectively bargain with employees [5, 94, 95, 97, 103, 104]. Emphasising the ubiquity of such measures, research found that almost one-third of letters of intent between the IMF and governments from 1998 to 2005 contained commitments towards flexible labour market regulation [105].

Examples abound. In a request for financial assistance from Morocco in 2011, the IMF emphasised the need for deregulating fixed-term contracts and reducing statutory labour protections [5]. Romania’s 2010 IMF programme targeted pensions with a 15% cut and required parliamentary approval of pension reform legislation, which included further pay-out reductions and raises to the retirement age [7]. The recent IMF programmes in Eurozone countries also incorporated labour-related reforms on deregulation and social security systems. Greece’s programme included reforms to the collective bargaining system—giving precedence to firm-level (as opposed to sectoral) agreements—and measures to reduce minimum wages and employee dismissal costs [3, 7]. Similarly, Portugal’s adjustment programme stipulated increases to the retirement age, weakening of collective bargaining, and the introduction of a public administration labour law aimed at aligning the public employment regime to the private sector rules and terminating tenure [7]. Furthermore, most Latin American governments made changes to their laws governing hiring, dismissal, and work hours in the past 20 years as a result of IMF pressure [104].

State-owned enterprise privatisation and pricing reforms can also directly impede the right to work. For the IMF and World Bank, state-owned enterprises are viewed as the major source of public deficit that, in turn, underpins many of the economic problems that sees countries turn to these institutions for loans [106]. Consequently, they have called for wide-ranging reforms of state-owned enterprises in order to reduce state aid and limit the deficit on the national budget. These reforms typically undermine labour rights by mandating reductions in entitlements for employees in state-owned-enterprises; they may also affect collective labour rights as collateral damage from the labour force’s diminishing bargaining power, given that the sector typically features high levels of collective representation. For example, in an IMF programme dating from 1993, Mongolia agreed to ‘establish new procedures of corporate governance’ for its state-owned enterprises, ‘[...] including requirements for fixed-term performance-based management contracts’ [107]. In Pakistan, privatisation of state-owned enterprises led to the replacement of stable jobs with precarious jobs involving sub-contractors, which were not fully subject to the labour law provisions [108].

Finally, several reforms can *indirectly* affect the protection of labour rights. Those related to the trade and exchange system frequently call for the liberalisation of trade, which prompts a race to the bottom in respect of labour rights as domestic producers pressure policy-makers to drive down labour costs in order to withstand global competition [94–96, 101, 109–111]. Furthermore, financial sector reforms that mandate a restrictive monetary policy may convince policy-makers to remove worker entitlements—such as a minimum wage—since higher wage levels tend to be inflationary [96].

### Civil and political rights

The responsibility of the state in respect of civil and political rights is also codified in the ICCPR. Political rights refer to the right to vote, the right to freedom of speech and the press, and the right not to be discriminated against on the basis of ethnicity, gender, sexual orientation, language, religion, social class, or political opinion; civil rights—also known as personal integrity or physical integrity rights—denote the right to be protected from torture, extra-judicial killings, disappearance, or political imprisonment, among others [94]. While there is less research on the civil and political rights consequences of structural adjustment reforms than on health and labour rights, most studies agree that the imposition of these policies exacerbates breaches of these human rights.

Arguments linking structural adjustment programmes to civil and political rights violations are rooted in two causal pathways. First, a direct pathway suggests that the transfer of power from the state to the market on the basis of conditionality can increase rights abuses by weakening the government’s ability to enforce such rights [94, 112–116]. The protection of civil rights, for example, requires government expenditures for properly trained and adequately compensated judges, police, and the military and for institutions to monitor the activities of enforcement entities [113]. One study also shows that IMF programmes are linked to deteriorating levels of respect for women’s rights because they undermine government ability and willingness to protect such rights [114]. In addition, cross-national studies find evidence that participation in structural adjustment programmes are associated with reductions in aggregated scores on borrowers’ levels of democracy [117, 118]. However, recent research contradicts these studies, observing a modest but positive effect of IMF programme participation on democracy. The study argues that autocratic regimes have less capacity to repress opposition when under a tighter budget constraint imposed by the IMF, allowing greater levels of political competition to emerge. Another study of 131 developing countries between 1981 and 2003 shows that while structural adjustment programmes led to increased hardship for the poor, greater civil conflict, and more repression of human rights, they are also—paradoxically—associated with some democratic reforms, including freer and fairer elections, greater freedom to form and join organisations, and greater freedom of speech and press [94].

Second, an indirect pathway suggests that the infringement of health and labour rights entailed by conditionality results in a rise of social protest and instability, leading to violent repression by the ruling government that amounts to breaches of civil and political rights. We know of several studies that test for—and support—this aggregate causal relationship [94, 112, 113, 119]. Cross-national empirical studies also support the notion that structural adjustment programmes are associated with increases in social and political instability, including incidents of mass demonstrations, strikes, riots, conflict, and coup d’états [120–124]. Furthermore, structural adjustment programmes are linked to declines in economic growth [117, 125–127], which is—in turn—associated with reduced respect for human rights [94, 128–130]. Case studies also typify the purported pattern of events [94, 131–135]. In Bolivia, limited progress under a 15-year stint of successive structural adjustment programmes came to a head in the early-2000s, following increases in unemployment and poverty, reductions in real wages, cuts in social expenditures, and—most infamously—the privatisation and subsequent price hikes of the city of Cochabamba’s water system [94]. Those most adversely affected responded with a spate of militant anti-government demonstrations and protests, to which the government responded by declaring a state of emergency and increasing its use of force against protestors. In Turkey, social unrest followed an announcement that the democratically elected government had signed an IMF programme in June 1980 and, by September of that year, the military dissolved parliament and suspended civilian political institutions [136].

## Conclusions

In offering loans in exchange for policy reforms, the IMF and World Bank are imbued with substantial policy influence on governments throughout the world. We have shown that since the introduction of structural adjustment in the 1980s, the two organisations have made ongoing attempts to overhaul the underlying institutions of borrowing countries, particularly via stabilisation, liberalisation, deregulation, and privatisation conditions. Our review revealed that conditionality prompts mostly perverse implications for the enjoyment of health, labour, and civil and political rights.

We note some caveats to our review. First, studies do not distinguish between the human rights effects of policy reforms actively sought by borrowing governments for inclusion in their lending arrangements from those unilaterally imposed by IFIs [13]. This gap offers an avenue for future research to pursue. Second, many of the studies deploy quantitative techniques to calculate average treatment effects on data spanning a period of a decade or more. Such a research design implicitly assumes that the impact on human rights is constant throughout the period considered, which may not be an accurate reflection of reality. That being said, there is also evidence of a startling degree of similarity in terms of the underlying market-liberalising impetus of structural adjustment across time [7].

While health rights have clear implications for public health, less obvious may be the public health implications of breaches to labour and civil and political rights. Structural adjustment is linked to diminishing labour rights, in turn linked to public health in terms of the extent to which people have access to decent work, a social determinant of health. Those without decent work and working conditions—that is, adequately paid, relatively stable, properly regulated, and with collective representation—typically experience a cascade of detrimental mental and physical health consequences over the life-course [6, 80–82]. So, too, it is the case that civil and political rights act as a (more distant) social determinant of health. Structural adjustment is linked to an increasing likelihood of civil conflict, in turn linked to violent repression which typically results in injury and causalities; unchecked civil conflict can also lead to physical destruction of health facilities, and, in its worst form—such as a full-fledged civil war—the total collapse of a nation’s health system [137, 138].

Responding to criticism in 2001 that IFIs were ignoring the human rights consequences of their activities, an IMF spokesperson stated that they do not have a mandate to promote human rights and are not ‘bound by various human rights declarations and conventions’ [139]. Indeed, it appears these organisations try to avoid human rights parlance altogether. According to a speechwriter of the IMF’s current Managing Director, Christine Lagarde, ‘You cannot put human rights in a speech, [or] it’ll be taken out’ [140]. Similarly, a United Nations assessment of World Bank policies provocatively concluded that the organisation is ‘a human rights-free zone.… It treats human rights more like an infectious disease than universal values and obligations’ [141]. But with the highly salient human rights violations in Greece’s structural adjustment programmes in recent memory [142], the Committee on Economic, Social and Cultural Rights issued a statement expressing that IFIs are ‘bound to comply with human rights … that are part of customary international law or of the general principles of law, both of which are sources of international law’ [143]. Since it is a legal responsibility of the IMF and World Bank to uphold human rights obligations, the question remains as to how the lending practices of these organisations might be revamped in ways that ensure they respect human rights.

Looking forward, the mechanisms identified in this review should serve as a guide for recalibrating structural adjustment programmes in ways that protect and respect existing human rights. In particular, future programmes should be designed with human rights as a core consideration. This entails a shift from *managing* negative human rights effects caused by conditionality—for instance, via poorly enforced social and priority spending targets—to *avoiding* policies that pose risks to human rights. As a corollary of this, a human rights impact assessment should be carried out before commencing a programme. It should involve consultation with relevant stakeholders, including public health experts, trade unions, the ILO, and the UN Human Rights Council. Programmes should also be regularly reviewed and evaluated not just in relation to their economic and fiscal targets, but against social policy targets, including reducing unemployment, poverty and social exclusion, and in terms of increased access to affordable healthcare.

In practice, this means an outright rejection of austerity policies, which have gone largely unchallenged despite the historical record of failure in spurring economic growth [144]. Indeed, analyses show that government spending in health and social protection not only improves the protection of health rights, but also boosts economic growth [75, 144]. In essence, there is no sound economic logic for the austerity policies pushed by the IMF and World Bank, while austerity represents an immediate threat to global health [145]. But it will also require wide-ranging reforms at the supranational level to ensure struggling governments are vested with the resources necessary to fund a comprehensive social protection system. In this regard, reform of the global taxation system, which currently allows multinational corporations and wealthy individuals to game the system to avoid paying taxes, represents the likeliest source of future revenues for struggling governments [144, 146].

What is clear is that improvements in public health will follow a concerted effort to meet internationally agreed-upon human rights obligations. It is therefore time for IFIs like the IMF and World Bank to take this prescription seriously.

## Abbreviations

ICCPR: International Covenant on Civil and Political Rights
ICESCR: International Covenant on Economic, Social, and Cultural Rights
IFI: International financial institution
ILO: International Labour Organisation
IMF: International Monetary Fund
UN: United Nations
